# Spontaneous rupture of hemorrhagic hepatic cyst: two case reports

**DOI:** 10.1186/s40792-022-01382-0

**Published:** 2022-02-21

**Authors:** Ichiya Chogahara, Akihiko Oshita, Hideki Nakahara, Toshiyuki Itamoto

**Affiliations:** 1 Department of Gastroenterological Surgery, Hiroshima Prefectural Hospital, 1-5-54 Ujina-Kanda, Minami-ku, Hiroshima, 7348530 Japan; 2 Department of Gastroenterological and Transplant Surgery Applied Life Sciences Institute of Biomedical and Health Sciences, Hiroshima University, 1-2-3 Kasumi, Minami-ku, Hiroshima, 7348551 Japan

**Keywords:** Hemorrhagic hepatic cyst, Spontaneous rupture, Laparoscopic deroofing, Sudden onset

## Abstract

**Background:**

Spontaneous rupture of a hemorrhagic hepatic cyst is extremely rare. There is no standard treatment recommended for this condition. We report two cases of hemorrhagic hepatic cysts that spontaneously ruptured and were successfully treated with laparoscopic deroofing. We review the literature and discuss the characteristic features of spontaneous rupture of hemorrhagic hepatic cysts and their treatment.

**Case presentation:**

The first patient was an 85-year-old man admitted for sudden-onset right hypochondralgia and fever. Computed tomography revealed a 13-cm hepatic cyst occupying the right lobe of the liver and spontaneous rupture of the cyst. Laparoscopic deroofing was performed and continuous oozing from the cystic wall was found. Histological examination revealed a simple hepatic cyst. The patient was discharged on postoperative day 6. In the second case, a 77-year-old woman who had been followed up for a simple hepatic cyst (13 cm) was admitted for sudden onset of right hypochondralgia. Computed tomography demonstrated a 9.9-cm hepatic cyst occupying segment 4 of the liver. Laparoscopic deroofing was performed and continuous oozing from the cystic wall was observed. Histological examination revealed a simple hepatic cyst. The patient was discharged on postoperative day 6.

**Conclusion:**

Laparoscopic deroofing was performed in patients with spontaneous rupture of hemorrhagic nonparasitic hepatic cysts.

## Background

Nonparasitic hepatic cysts (NPHCs) are the most common diseases of the liver. Most patients with NPHC are asymptomatic and do not require treatment, while those with large cysts might be symptomatic and require treatment. Spontaneous rupture of hemorrhagic NPHC is extremely rare. There is no standard recommended treatment for a ruptured hemorrhagic NPHC. Herein, we report two rare cases in which laparoscopic deroofing was performed to treat spontaneous rupture of a hemorrhagic NPHC.

## Case presentation

### Case 1

An 85-year-old man who had been followed up for a simple hepatic cyst (89 × 85 mm) for 2 years was referred for sudden-onset right hypochondralgia and fever. He had no history of previous surgery and was a known case of hypertension. The patient was not on anticoagulants. The onset of pain was spontaneous, and there was tenderness in the right upper quadrant without guarding; there was no history of trauma. Laboratory test results revealed mild anemia (Table 1).

**Table 1:** Blood examination on arrival

WBC	8100/μL	T-Bil	0.6 mg/dL	TP	7.0 g/dL
RBC	4,170,000/μL	AST	27 U/L	Alb	4.3 g/dL
Hb	13.1 g/dL	ALT	23 U/L	BUN	17.7 mg/dL
Hct	37.7%	ALP	298 U/L	Cr	0.94 mg/dL
Plt	149,000/μL	γ-GTP	53 U/L	Na	140 mEq/L
		LDH	191 U/L	K	3.8 mEq/L
APTT	31.3 s	ChE	217 U/L	Cl	107 mEq/L
PT-INR	1.11	BS	116 mg/dL	CRP	0.9 mg/dL

*WBC* white blood cells, *RBC* red blood cells, *Hb* hemoglobin, *Hct* hematocrit, *PLT* platelets, *APTT* activated partial thromboplastin time, *PT-INR* prothrombin time-international normalized ratio, *T-Bil* total bilirubin, *AST* aspartate aminotransferase, *ALT* alanine transaminase, *ALP* alkaline phosphatase, *γ-GTP* γ-glutamyltranspeptidase, *LDH* lactate dehydrogenase, *ChE* cholinesterase, *TP* total protein, *Alb* albumin, *BUN* blood urea nitrogen, *Cre* creatinine, *Na* natrium, *K* kalium, *Cl* chlorine, *BS* blood sugar, *CRP* C-reactive protein

**Fig. 1:**
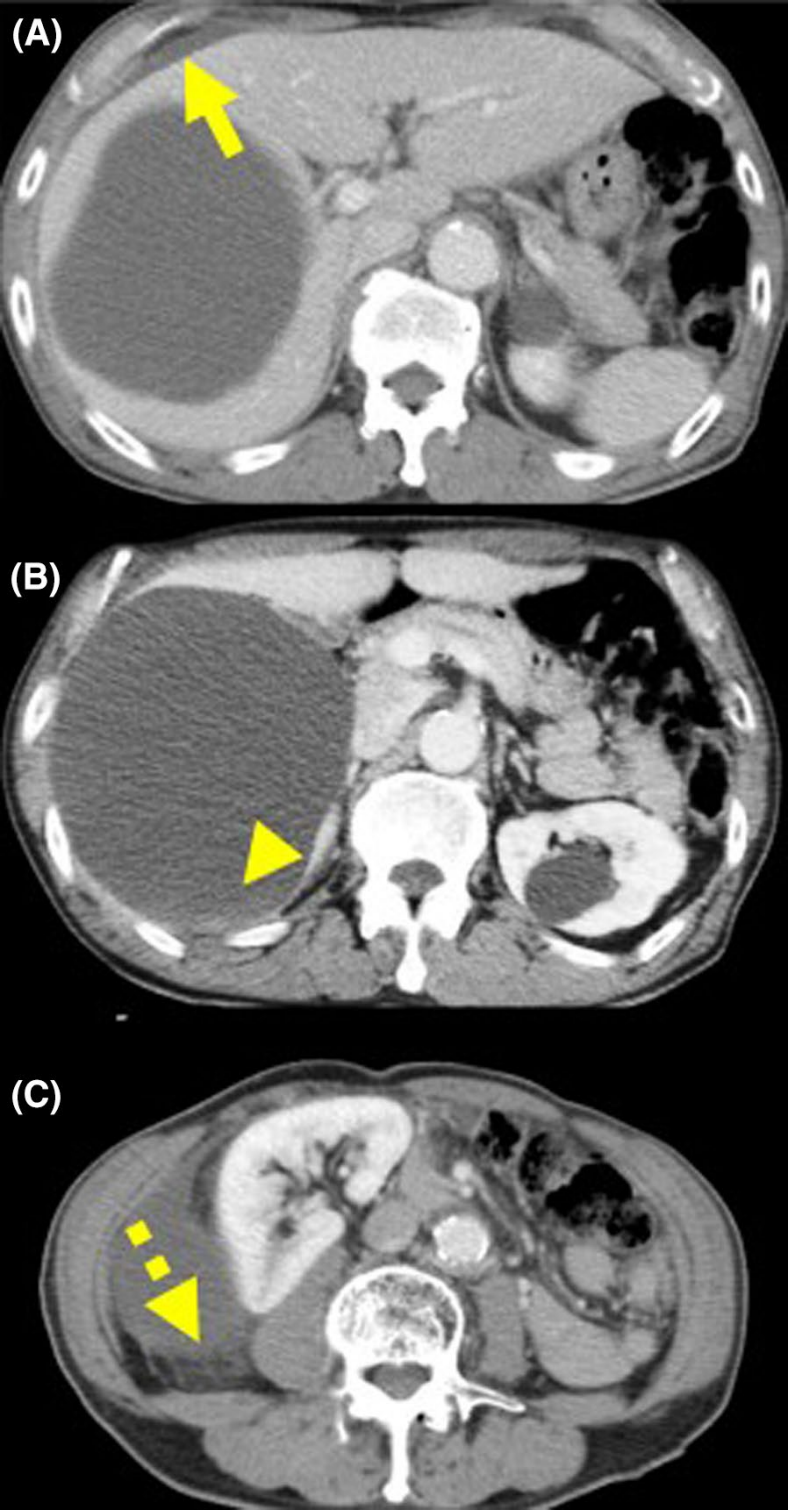
Enhanced CT of case 1. **A** CT demonstrated a simple hepatic cyst occupying the right lobe of the liver, and fluid collection on the surface of the liver (arrow). **B** The Hounsfield Unit level was 40 at the lower level of the cyst (arrowhead). **C** A part of the cystic wall was not smooth but serrated (dotted arrow)

Computed tomography (CT) showed a simple hepatic cyst 13 cm in diameter, occupying the right lobe of the liver. The cystic wall was not smooth but serrated. The Hounsfield Unit level was 40 at the lower level of the cyst and less than 10 at the upper level. Fluid collection was observed on the liver surface (Fig. 1). These findings indicated a ruptured cyst and intracystic hemorrhage. Following the diagnosis and considering stable vital signs and no symptoms of peritonitis, a semi-urgent elective surgery was scheduled. However, the hemoglobin level rapidly decreased from 13.1 g/dL to 11.2 g/dL on the following day. Therefore, urgent laparoscopic intervention was performed.

**Fig. 2:**
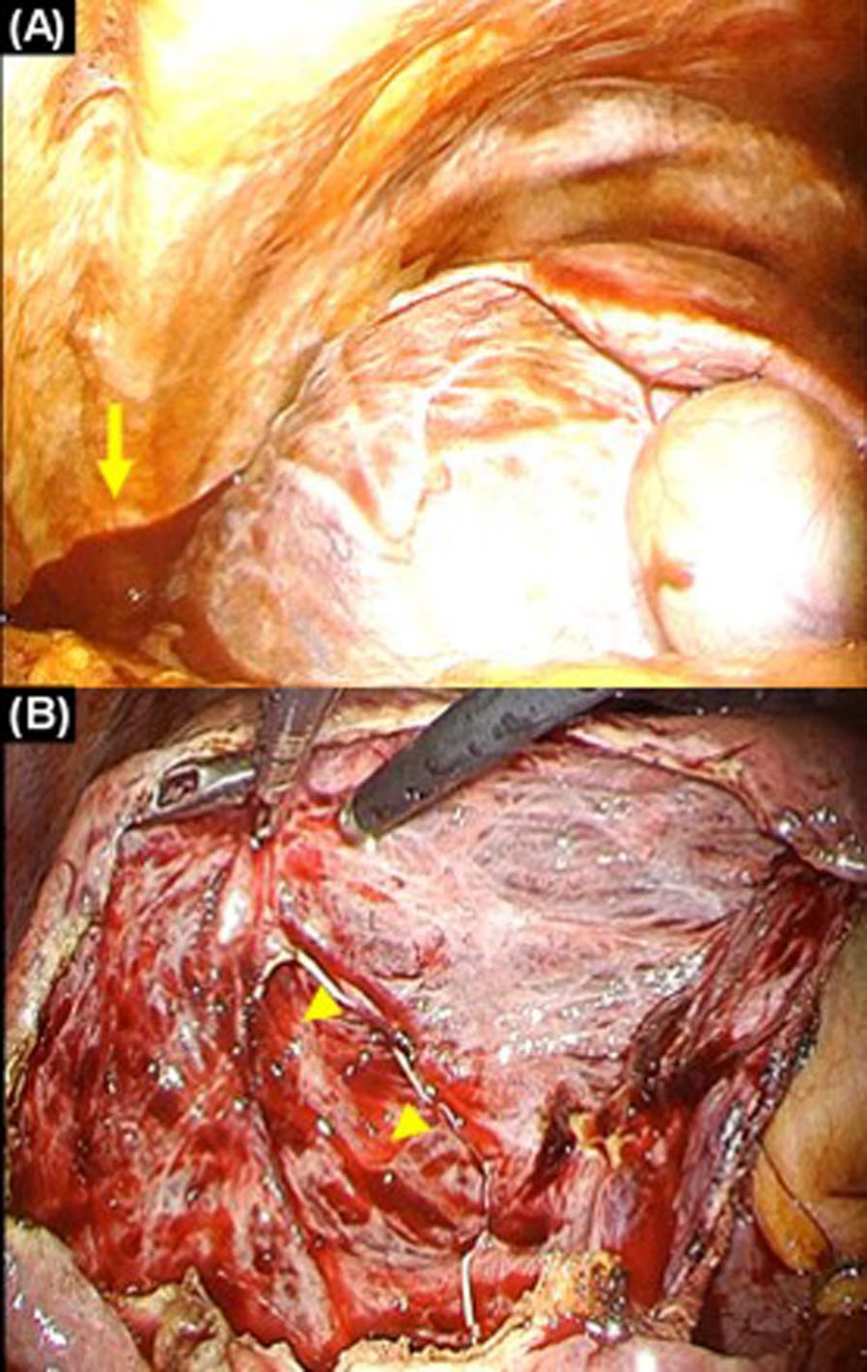
Intraoperative findings of case 1. **A** Surgical exploration revealed hemoperitoneum (arrow) and a voluminous hepatic cyst, occupying the right lobe. **B** Continuous oozing was found at the posterior wall of the cyst (arrow heads)

Abundant hemoperitoneum and a voluminous hepatic cyst occupying the right lobe were observed through laparoscopy. The anterior wall of the cyst was deroofed and resected (Fig. 2). Approximately 1 L of partially coagulated blood was removed. After evacuation, continuous oozing which might be venous was found at the posterior wall of the cyst, and hemostasis was confirmed after cauterization. Results of the laboratory tests of the cystic fluid showed a normal level of total bilirubin. No bacteria or malignant cells were identified in the cystic fluid. Pathological investigation revealed no evidence of malignancy or *Echinococcus* species infection. The postoperative course was uneventful, and the patient was discharged on postoperative day 6.

### Case 2

A 72-year-old woman, who had been followed up for a simple hepatic cyst (13 cm) for 3 years was referred for sudden onset of right hypochondralgia. She had previously undergone partial mastectomy and was treated for hypertension and hyperlipidemia. She had no history of anticoagulant drug intake or trauma. The onset of pain was spontaneous and there was tenderness in the right upper quadrant, with no guarding. Results of the laboratory tests revealed elevated WBC and CRP levels (Table 2).

**Table 2:** Blood examination on arrival

WBC	10,500/μL	T-Bil	0.5 mg/dL	TP	6.2 g/dL
RBC	3,990,000/μL	AST	22 U/L	Alb	3.5 g/dL
Hb	12.7 g/dL	ALT	17 U/L	BUN	14.0 mg/dL
Hct	37.2%	ALP	280 U/L	Cr	0.55 mg/dL
Plt	245,000/μL	γ-GTP	30 U/L	Na	142 mEq/L
		LDH	230 U/L	K	3.8 mEq/L
		ChE	248 U/L	Cl	116 mEq/L
PT-INR	1.03	BS	116 mg/dL	CRP	1.08 mg/dL

*WBC* white blood cells, *RBC* red blood cells, *Hb* hemoglobin, *Hct* hematocrit, *PLT* platelets, *APTT* activated partial thromboplastin time, *PT-INR* prothrombin time-international normalized ratio, *T-Bil* total bilirubin, *AST* aspartate aminotransferase, *ALT* alanine transaminase, *ALP* alkaline phosphatase, *γ-GTP* γ-glutamyltranspeptidase, *LDH* lactate dehydrogenase, *ChE* cholinesterase, *TP* total protein, *Alb* albumin, *BUN* blood urea nitrogen, *Cre* creatinine, *Na* natrium, *K* kalium, *Cl* chlorine, *BS* blood sugar, *CRP* C-reactive protein

**Fig. 3:**
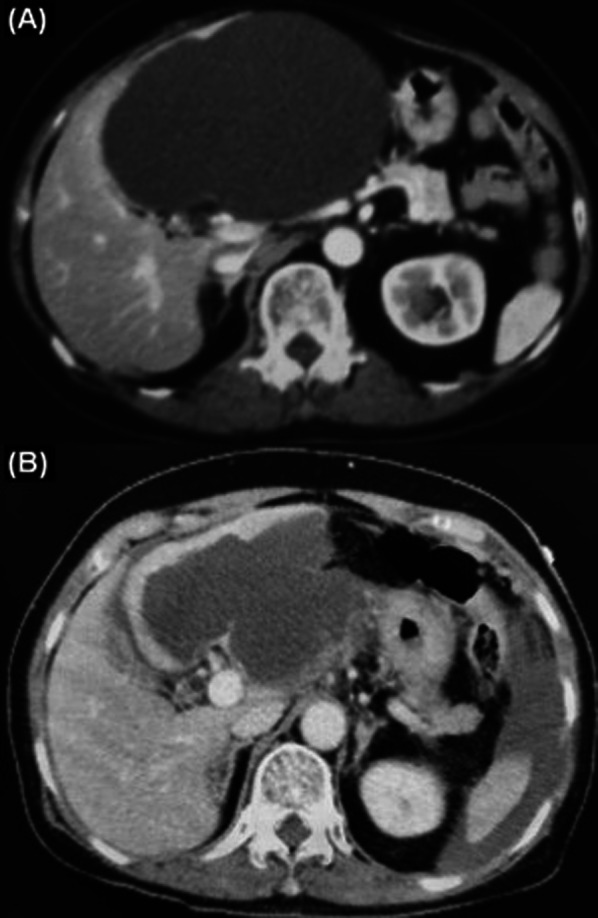
Enhanced CT of case 2. **A** CT demonstrated a simple hepatic cyst (13 cm) in segment 4 previously. **B** CT showed ruptured cyst (9.9 cm) and fluid collection between the spleen and the abdominal wall

CT demonstrated a simple hepatic cyst 9.9 cm in diameter occupying segment 4 of the liver. Fluid accumulation was seen between the spleen and abdominal wall (Fig. 3); the diagnosis was a ruptured cyst. Following the diagnosis, stable vital signs, and no symptoms of peritonitis, a semi-urgent surgery was scheduled.

Two days later, laparoscopic deroofing was performed. Hemoperitoneum and a hepatic cyst occupying segment 4 of the liver were observed through the laparoscope. The anterior wall of the cyst was deroofed and resected. Approximately 470 mL of partially coagulated blood was removed. After evacuation, continuous oozing which might be venous was observed at the posterior wall of the cyst, and hemostasis was confirmed after cauterization. Results of the laboratory tests of the cystic fluid showed a normal level of total bilirubin. No bacteria or malignant cells were identified in the cystic fluid. Pathological investigation revealed no evidence of malignancy or infection. The postoperative course was uneventful, and the patient was discharged on postoperative day 6.

## Discussion

Intrahepatic cysts are generally classified as congenital, traumatic, inflammatory, parasitic, or neoplastic [1]. NPHC is the most common disease of the liver and is found in approximately 1–5% of the general population [2]. The female-to-male ratio is 3:1. Most patients with NPHC are asymptomatic and do not require treatment. However, patients with symptoms, such as appetite loss due to compression of adjacent structures, abdominal pain, intracystic infection, jaundice, cyst–biliary communication, and rupture, which require treatment [3].

Although rupture of hepatic cysts is sometimes seen in cases of infection with *Echinococcus* species, spontaneous rupture of a hemorrhagic NPHC is extremely rare [4]. A search of English-language reports published in PubMed using the keywords ‘liver’, ‘hepatic’, ‘cyst’, ‘rupture’, ‘spontaneously’, and ‘hemorrhage’ from 1999 to 2021 returned only 14 cases that described spontaneous rupture of hemorrhagic NPHC, including our cases (Table 3) [5–16]. Since the cystic lesions of autosomal dominant polycystic kidney disease (ADPKD) have different pathogeneses, patients with ADPKD were excluded from the results of our keyword search. There were no differences between the sexes. Eleven patients were more than 60 years old. The chief complaints of 11 patients were abdominal pain, and four of them had peritonitis. Twelve patients had sudden-onset pain, including chest pain. The diameter of the cyst exceeded 10 cm in 12 patients. The final diagnoses were simple hepatic cysts in nine cases, biliary cysts in three cases, and bacterial infection in one case. In cases with sudden-onset pain during follow-up of an NPHC larger than 10 cm, rupture of a hemorrhagic hepatic cyst should be suspected.

**Table 3:** Summary of patients with spontaneously ruptured hemorrhagic hepatic cyst

Author	Year	Age	Sex	Chief complaint	Sudden onset	Peritonitis	Size (cm)	Treatment	Final diagnosis	Discharge	Recurrence
Yamaguchi	1999	61	M	Epigastralgia	+	+	13	Left trisegmentectomy	Simple cyst	POD 31	−
Ishikawa	2002	42	F	Discomfort in right hypochondrium	ND	−	10	TAE + percutaneous drainage laparotomy and cystectomy	Simple cyst	ND − > POD 12	+
Kanazawa	2003	78	M	Right hypochondralgia fever elevation	+	−	ND	Percutaneous drainage, antibiotics and ethanol injection	Bacterial infection	ND	−
Cheung	2005	73	F	Abdominal pain fever elevation	ND	+	15	Laparoscopic deroofing	ND	POD 4	−
Marion	2013	37	F	Right hypochondralgia hemorrhagic shock	+	−	18	Laparotomy and cystectomy	Biliary cyst	POD 6	−
Simon	2015	63	M	Right hypochondralgia	+	−	14	Conservative therapy	Simple cyst	Day 31	−
Hotta	2015	62	F	Right hypochondralgia	+	−	13	Percutaneous drainage and antibiotics injection	Simple cyst	Day 12	−
Inoue	2015	59	F	Abdominal pain	+	+	10	Laparotomy and deroofing	Simple cyst	POD 8	−
Wang	2015	71	M	Right hypochondralgia hemorrhagic shock	+	−	7.9	Conservative therapy	Simple cyst	Day 13	−
Vannucchi	2016	73	M	Right hypochondralgia	+	−	10	Laparotomy	Biliary cyst	POD 8	−
Tong	2019	70	F	Chest pain	+	−	13	Laparotomy	Simple cyst	POD 9	−
Amaral	2020	72	F	Right hypochondralgia, fever elevation	+	+	16	Laparotomy	Biliary cyst	POD 2	−
Our case	2021	85	M	Right hypochondralgia	+	−	13	Laparoscopic deroofing	Simple cyst	POD 6	−
Our case	2021	77	F	Right hypochondralgia	+	−	13	Laparoscopic deroofing	Simple cyst	POD 6	−

*M* male, *F* female, *TAE* transcatheter arterial embolization, *ND* not determined, *POD* postoperative day

Takahashi et al. reported that an increase in intracystic pressure induces necrosis in the cyst wall and consequent intracystic bleeding and rupture [17]. An increase in intracystic pressure might be related to the secretion of the cystic epithelium, exudation due to an infection, or intracystic bleeding. A fragile cyst wall due to invasion of cancer could also increase the risk of spontaneous rupture.

There is no standard strategy for the management of ruptured hemorrhagic NPHC. In general, the therapeutic options include surgical procedures and non-surgical procedures, such as transcatheter arterial embolization (TAE), percutaneous drainage, and sclerotherapy. Non-surgical management might be a useful option for patients with stable vital signs. However, high recurrence rates have been reported after non-surgical treatment in patients with symptomatic hepatic cysts [6, 18, 19]. In recent times, a laparoscopic approach has been proposed and is considered as a minimally invasive treatment. In our review, of the ten patients who underwent surgical treatment, three were treated using laparoscopy. Patients who underwent laparoscopic treatment were discharged earlier than those treated with other modalities and had no recurrence. Recurrence was observed in only one patient who underwent TAE and percutaneous drainage. While HCC is fed with the artery and TAE is effective for the ruptured HCC, a hemorrhagic hepatic cyst is caused by the damage of the cystic wall and bleeding is peripheral. It might be the reason why TAE was not effective.

In our review, 12 of 14 cases, including our case, had sudden-onset pain. Furthermore, two of them developed hemorrhagic shock and were in a life-threatening condition. Sudden-onset pain in patients with a history of hepatic cysts could be an indicator of a ruptured hemorrhagic cyst. These patients require imaging studies, including ultrasonography and CT. Dynamic CT with contrast agent is very useful to diagnose hemorrhage by showing the extravasation. However, in cases of hemorrhagic hepatic cysts, bleeding is not so active and not from the artery or the portal vein, but from the peripheral vein of the cystic wall. Thus, dynamic CT could not show the extravasation from the cystic wall in our cases. Emergency surgery should be considered in patients with unstable vital signs or peritonitis. In addition, we suggest that in patients with a decrease in hemoglobin concentration, such as in case 1, urgent surgery should be considered even when vital signs are stable. Elective surgery might be appropriate for patients without unstable vital signs, peritonitis, or progressive decrease in hemoglobin.

## Conclusion

Spontaneous rupture of a hemorrhagic NPHC is extremely rare. Laparoscopic deroofing was successfully performed in patients with spontaneous rupture of hemorrhagic NPHC.

## Data Availability

Not applicable.

## Abbreviations

NPHC: Nonparasitic hepatic cyst
CT: Computed tomography
CA19-9: Carbohydrate antigen 19-9
CEA: Carcinoembryonic antigen
PLD: Polycystic liver disease
TAE: Transcatheter arterial embolization
